# Social preferences trump emotions in human responses to unfair offers

**DOI:** 10.1038/s41598-023-36715-y

**Published:** 2023-06-13

**Authors:** Vincent Buskens, Ingrid Kovacic, Elwin Rutterkamp, Arnout van de Rijt, David Terburg

**Affiliations:** 1 Department of Sociology/ICS, Utrecht University, Utrecht, The Netherlands; 2 Department of Political and Social Sciences, European University Institute, Fiesole, Italy; 3 Department of Psychology, Utrecht University, Utrecht, The Netherlands

**Keywords:** Human behaviour, Emotion, Motivation, Social behaviour, Social neuroscience

## Abstract

People commonly reject unfair offers even if this leaves them worse off. Some explain this as a rational response based on social preferences. Others argue that emotions override self-interest in the determination of rejection behavior. We conducted an experiment in which we measured responders’ biophysical reactions (EEG and EMG) to fair and unfair offers. We measured biophysical *trait anger* using resting-state EEG (frontal alpha-asymmetry), *state anger* using facial expressions, offer *expectancy processing* using event-related EEG (medial-frontal negativity; MFN) and self-reported emotions. We systematically varied whether rejections led proposers to lose their share (Ultimatum Game; UG) or not (Impunity Game; IG). Results favor preference-based accounts: Impunity minimizes rejection despite increasing subjectively reported anger. Unfair offers evoke frowning responses, but frowning does not predict rejection. Prosocial responders reject unfair UG offers more often after unmet fairness expectations. These results suggest that responders do not reject unfairness out of anger. Rather, people seem motivated to reject unfair offers when they violate their behavioral code but only when rejection has payoff consequences for the proposer, allowing them to reciprocate and restore equity. Thus, social preferences trump emotions when responding to unfair offers.

## Introduction

People are willing to reject unfair deals even if they are worse off without a deal. In experimental studies of negotiation, participants routinely decline final offers that are better than nothing. There are two main theoretical accounts of the rejection of unequal divisions of a resource. First, people may have *social preferences* over payoffs incurred by others. Individuals may reject offers because they prefer that bad behavior is punished – reciprocity^1,2^ – or that outcomes are more equitable – inequity aversion^3^. In this account, preferences dominate emotions: where they conflict, preferences determine behavior. Second, unequal proposals may evoke *emotions* such as anger that *override self-interest,* including possible social preferences, in the determination of behavior^4^. Such emotion-driven behavior could have an evolutionary basis as a commitment device that induces fair treatment in others^4,5^. The two accounts fundamentally differ on whether the rejection of unfair offers should be viewed as rational, producing the desired outcome, or as an irrational response that ignores the consequences of the rejection.

To empirically investigate the origins of rejection behavior, studies have focused on various versions of a two-person game in which a proposer offers a division of a resource. In the ‘Ultimatum Game’ (UG)^1,6–8^, the responder can accept the offer or refuse it, leaving both players with nothing. In the ‘Dictator Game’ (DG)^9^ responders must accept any proposed split. In the less often studied ‘Impunity Game’ (IG)^4,10,11^ responders can refuse their share but proposers keep their share regardless. Laboratory experiments show that proposed offers in the UG are more equitable than in the DG, suggesting that proposers indeed anticipate either social preference- or emotion-based rejections by responders. A recent meta-analysis^12^ illustrates that rejection behavior in the UG indeed relates to social preferences as well as aggression. Yet the evidence fails to clearly adjudicate between social preferences and emotions as the driver of behavior.

Preference-based accounts predict greater rejection of unfair offers in the UG than in the IG: While rejecting unfair offers decreases inequity in the UG, it increases inequity in the IG, and while rejecting unfair offers achieves punishment in the UG, it fails to do so in the IG^13^. Because rejection in the IG worsens inequity and fails to punish proposers, it can only be explained by emotional reactions trumping self-interest, and not by social preferences. In many UG experiments, the most commonly proposed offer is a 50–50 split and around half of the responders reject unfair offers in which they would receive less than 30% of the total sum^14^. Findings on the less commonly studied IG are mixed. Some find that in contrast to the UG, unfair offers in the IG are commonly made and hardly ever rejected^13,15^, supporting a preference-based account. Others find rejection rates in the IG that are comparable to the UG^4,11^, supporting an emotion-based account. The explanation in Yamagishi et al.^4^ for the difference is that in Bolton and Zwick^13^ the accept/reject terminology is not used in the experimental design. Here we will stick to that terminology. Disentangling the preference- and emotion-based accounts is the purpose of this paper.

There are two key challenges in past empirical studies that complicate inference from prior results, both of which we aim to overcome in the present study. The first is that proposer behavior in real interaction studies is different in UG and IG, so differences in responder behavior are uncontrolled. For example, the experimental setup in Bolton and Zwick^13^ does not guarantee constant unfair offers across games, allowing unfair offers to become expected in the IG, and responders to adapt to it by not rejecting the offer. Conversely, in the UG, offers are more often fair, which may set an expectation for more fair offers, leading to more rejection of unfair offers. One workaround that guarantees equal prevalence of unfair offers is using preprogrammed offers, but this deception may undermine the credibility of proposer behavior. E.g. the experimental set-ups of the three studies in Yamagishi et al.^4^ use the strategy method (study 1), only providing unfair offers to responders (study 2) and providing predefined numbers of unfair offers (study 3). Although participants in some cases might be convinced that they directly play with a proposer, such deviations from natural human-to-human interaction may affect responders’ behavior if they rightly doubt the actual proposer payoff consequences of their own decisions. The second limitation of past work is the limited measurement of emotions and thought processes. Those studies that do make use of direct biophysical measures, such as electroencephalography (EEG) or functional magnetic resonance imaging (fMRI)^4,7,16–19^, use preprogrammed offers for balancing unfair offers, because many trials with unfair offers are needed for good EEG and fMRI measurement, especially when rejection is uncommon.

The present experimental study overcomes both limitations, achieving equal levels of unfair offers while still having genuine and direct interactions between participants and also drawing on a range of biophysical measures. Besides self-reported emotions, we record brain activity through EEG and facial expressions through electromyography (EMG) in real-time human-to-human negotiation. We test preference- and emotion-based theories by comparing social preferences, emotions, and rejection behavior in UG and IG. This allows us to answer three questions:

1. *Does responder’s behavior vary with the ability to impact proposer’s payoff?*
2. *How do responder’s emotional and biophysical reactions depend on proposer’s behavior, the ability to impact proposer’s payoff, and social preferences of responders?*
3. *To what extent do social preferences or emotions drive rejection behavior?*

We ran 10 sessions in which we invited 14–18 participants to our lab, half proposers and half responders. We derived participants’ social preferences from a standard measurement of social value orientation. All responders self-reported on emotions experienced when faced with fair and unfair offers in UG and IG, allowing within-participant comparisons across offers and game types. Four of the responders received an EEG cap and electrodes for EMG measurement (see “Methods” for further details). Proposers and responders knew they were randomly and anonymously matched for 48 games and that they would not be able to recognize each other if they met again in later games. The only feedback on game outcomes that proposers received was after finishing all 48 games, namely the total number of points they earned. That this was the only feedback they would receive was common knowledge. This prevented proposers from changing their behavior in reaction to prior rejection behavior by responders. It also prevented responders from communicating their concerns about an unfair offer to proposers by means of rejecting the offer and from establishing a reputation for being tough.

Participants played 12 periods of one game (UG or IG); subsequently, 12 periods of the other game; then again 12 periods of the first game and they finished with 12 periods of the game they did not start with. In each period, a pair of participants thus played either a UG or IG. After each period, proposers and responders were randomly rematched to a participant in the other role in the laboratory. This setup was common knowledge for all participants, but only responders knew whether they started in the UG or the IG. Therefore, the proposer faced a 50% chance that if the offer was rejected (UG) proposer would receive nothing and a 50% chance proposer would then keep the proposer’s share (IG). The proposer had only two options: a fair offer (50:50) or a specific unfair offer to their own advantage (80:20). This setup ensured there was no systematic difference in the rate of unfair offers between UG and IG because the proposer could not differentiate. This is important because it prevents responders from holding different expectations about proposer behavior in UG and IG that could otherwise confound social preferences as the cause of differential rejection behavior.

Our inclusion of other participants in addition to the four EEG/EMG-equipped participants has two key advantages. First, it keeps the number of interactions between the same two participants limited across a large number of periods, preventing responders from obtaining many offers from the same proposer. Second, comparisons between EEG/EMG-equipped responders and other responders can evaluate whether being connected to these measurement instruments itself affects behavior or self-reported emotions, e.g. because of physical discomfort or psychological impact.

Although our UG/IG manipulation tests specific hypotheses with respect to the *emotional commitment* versus *social preference* debate, it does not measure these constructs directly. Therefore, we also measured self-reported social value orientation and emotion as well as biophysical reactions to the offers that capture these constructs implicitly. Particularly, the latter measures can provide valuable information as they reflect largely automatic reactions to (un)fairness that precede the decision to accept or reject the offer. We thus aim to use these biophysical measures to substantiate our arguments with respect to the *emotional commitment* versus *social preference* debate and focus on three potential predictors of rejection behavior: First, as a state measure of *social preferences,* we measure the EEG’s event-related negativity on medial-frontal electrode locations averaging the signal from 260 to 300 ms after an offer is presented, i.e. the medial-frontal negativity (MFN). The MFN is argued to result from anterior cingulate cortex (ACC) activity reflecting conflict monitoring and expectation processing about an event. It is most pronounced in response to unfair offers, particularly in responders with high concerns for fairness^19,20^. In other words, prosocial individuals show a stronger MFN to unfairness because unfairness does not match their social expectation of proposer behavior. Thus, if *social preferences* dictate the response to unfairness, we expect that the MFN to unfairness is stronger in prosocial individuals and that this MFN predicts rejection behavior. To control the MFN for attentional modulation we also evaluate the P2, an event-related positivity on medial-frontal electrode locations preceding the MFN around 200 ms following the offer. Increased P2 amplitudes reflect increased attention to a stimulus, and fairness and social preferences have both been shown to affect the P2 in similar ways as the MFN^19^. Due to the opposite polarity and the fact that the P2 precedes the MFN, expectancy processing effects on rejection behavior as indexed by the MFN can potentially be due to attention processing effects as indexed by the P2. Therefore, we also assessed P2 activity on the same electrode locations as the MFN. MFN and P2 measures were averaged over trials by game type and by fair and unfair offers to ensure that this predictor has a reliable signal-to-noise ratio.

Second, as a state measure of *emotional commitment* we measure the ‘frown’ (corrugator supercilii) and ‘smile’ (zygomaticus major) muscles using facial EMG. Even without overtly expressed facial emotions, these muscles have been shown to reflect emotional reactions to unfairness^21^. Thus, if *emotional commitment* dictates the response to unfairness, we expect that unfairness evokes an angry frowning response, which in turn would predict rejection behavior.

Third, as a trait measure of *emotional commitment,* we assess frontal alpha-asymmetry (FAA), which is a biophysical index of trait anger. The power of alpha-frequencies (8–12 Hz) in the resting-state EEG is indicative of cortical idling, which can be used as an inverse predictor of hemispheric dominance. Left-sided frontal dominance, as indexed by the right-sided asymmetry in alpha-power, is used as a measure of proneness to anger, aggression, and approach motivation in correlational^22^ as well as brain stimulation studies^23^. If *emotional commitment* dictates the response to unfairness, we expect that individuals with high trait anger as indexed by FAA reject unfairness more often. In combination, these biophysical measures capture the emotional as well as social preferential reactions to (un)fairness preceding the decisions to reject or accept (un)fair offers.

The standard game-theoretic prediction assuming no social preferences is that responders accept any positive offer in the UG as well as the IG. The accumulated evidence we reviewed suggests that this prediction will be rejected. Our aim is to evaluate existing theoretical accounts for the rejection of unfair offers. First, in line with our first question, we evaluate whether reaction behavior varies between UG and IG. Here the social preference account claims that rejection of unfair offers should predominantly occur in the UG and in particular for participants with prosocial preferences because they prefer more equal outcomes and they can obtain that by rejecting unfair offers in the UG**.** For the IG, this account predicts that offers should not be rejected because that only further increases inequity. The emotional commitment account, however, states that emotions can overrule preferences. Unfair offers will induce anger in UG and IG and if emotional commitment dominates behavior one expects similar rates of rejection of unfair offers in both games.

Concerning our second question, note that the social preference account does not preclude that people react emotionally if others act against their social preferences or what they think is appropriate behavior. On the contrary, the strength of the emotions may even be an indicator of how strong the preferences are^16^. Therefore, we do expect that more prosocial participants will be angrier with unfair offers than less prosocial participants. They might even be angrier in the IG than in the UG because they cannot repair the proposed inequality. As explained above, the MFN reaction to unfairness of prosocial participants is also expected to be stronger. The two theoretical accounts do not predict differences in the drivers of the emotional and biophysical reactions, but merely in the consequences of these reactions for behavior. This is the topic of the third research question.

The social preference account states that there will be only a limited effect of the emotional reactions on behavior in particular if the behavior would go against the preferences. In addition, it predicts that MFN, measuring an offer’s perceived violation of social expectations, will have an effect on rejection behavior. However, it does not predict frowning, state anger, and self-reported anger to impact behavior directly. By contrast, the emotional commitment account predicts frowning, state anger, and self-reports of anger to render rejection of unfair proposals more likely.

The results of the paper are organized as follows. First, we show that the behavior in games in combination with the self-report measures is in line with a social preference explanation of rejection behavior. Then, we show that biophysical reactions to unfair offers differ between IG and UG as well as between more prosocial and more individualistic participants. Finally, we show that we can predict rejection behavior with the MFN measurement of social preferences, but not with the EMG measurement of emotional commitment.

## Results

### Rejection behavior

In our experiment, participants turn out to reject unfair offers substantially more often in UG (33.3%) than in the IG (6.5%). (Table S1.4, see Tables S1.1 through S1.5 for full descriptives of proposer and responder behavior crossed with social value orientations). This difference is highly significant (*p* < 0.001) and does not vary between participants with and without biophysical measurements (Table S2.1). This result provides strong support for the social preference account and goes against the emotional commitment account. (Choices of statistical tests are motivated in the Methods section, while the complete statistical results are provided in the SI). The result replicates findings from other studies with very low rejection rates in the IG^13,15^, and goes against theoretical arguments from others^4,11,24^. The result is independent of whether or not responders are connected to biophysical measurement instruments. Also, offers do indeed not significantly differ between UG and IG, as induced by our design, because proposers are not informed about whether they play UG or IG.

While the rejection-minimizing effect of proposer immunity falsifies the emotional commitment argument, it does not rule out the possibility that emotions are nonetheless the primary driver of rejection behavior. Emotions may be stronger in the UG than in the IG, for example, if unfair offers are for some reason experienced as less upsetting when rejection has no consequences for the responder. Both emotions and social preferences are hence still potential drivers of rejection behavior, and measures of both are needed to empirically isolate their effects.

### Explaining emotions and biophysical responses

Participants self-report (post hoc) that they experience rather low levels of anger and envy when receiving unfair offers (see Table S2.2). The highest average anger score on a 0 to 4 scale is 1.56 being in between ‘a little’ and ‘moderately’ is scored for unfair offers in the IG. Pairwise *t* tests show that participants report being less angry, less envious, and happier with unfair offers in the UG than with unfair offers in the IG (all tests with *p* < 0.001), while the comparison of fair offers does not show large systematic differences (see Table S2.2). This implies that not being able to impact the proposer’s payoff through rejection makes participants *feel worse* about receiving an unfair offer while at the same time making them *more likely to accept* it. These more negative emotion ratings may be borne out of frustration for not being able to punish. These observations go against the notion of emotions dominating in the determination of rejection behavior: Despite anger being greater in the IG, rejection is much rarer.

**Figure 1:**
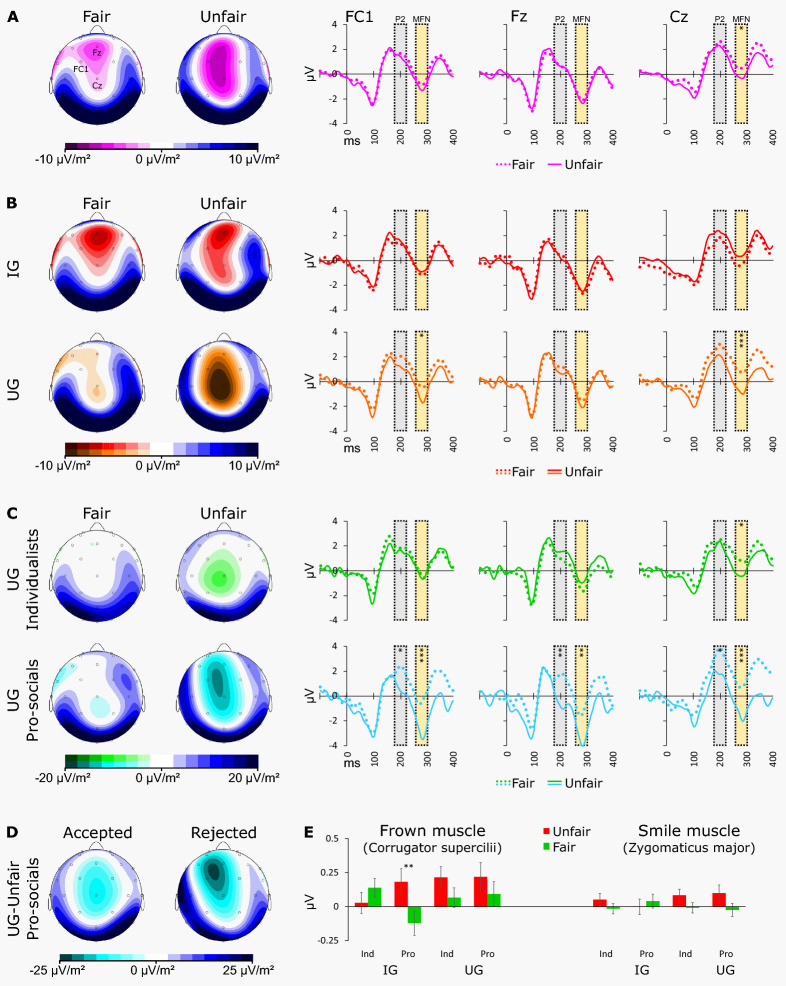
Overview of how game type, fairness, social preferences, and responder actions vary with biophysical responses to offers. Panels (**A**–**C**) show a step-by-step breakdown of the three-way interaction of offer (fair/unfair), game (UG/IG) and social preference (prosocial/individualistic) on the MFN. Presented are event-related waveforms (P2 and MFN on the 10/20 EEG locations FC1, Fz, and Cz) in response to the offers showing the statistically relevant effects, and current source density (CSD) maps to visualize an estimation of the location of the underlying source in the brain. Panel (**A**) Waveforms across both games: The MFN is stronger in response to unfair compared to fair offers. Panel (**B**) Waveforms for UG and IG offers separately: Only in the UG is the MFN stronger in response to unfair compared to fair offers. Panel (**C**) Waveforms separately for prosocial and individualistic responders to UG offers: Only in prosocial responders are the P2 and MFN stronger in response to unfair compared to fair UG offers. Panel (**D**) CSD map illustrating the amplification and left-frontal shift of the MFN in prosocial responders after receiving unfair UG offers that are accepted versus those that are rejected. Panel (**E**) Estimated marginal means with standard errors of the averaged EMG-activity (2 s) of the ‘frown’ (corrugator supercilii) and ‘smile’ (zygomaticus major) muscles in response to fair and unfair offers in the IG and UG for individualistic (Ind) and prosocial (Pro) responders. Only for prosocial responders in the IG do we see that unfair offers evoke more frowning. Significance levels come from two-level regression models with asterisks indicating the significance of the relevant comparisons: *p < 0.05, **p < 0.01, ***p < 0.001 (see Tables S2.6–S2.11). All CSD maps are based on the average signal between 260 and 300 ms after the offers, which is also the interval used for the analyses of the MFN, indicated with yellow areas in the waveform graphs.

We now analyze biophysical reactions to offers using two-level regression models, confirming their validity, establish baseline effects of game type and fairness, and evaluate how they relate to social value orientation. For our EEG measures, we focus on the event-related negativity on medial-frontal electrode locations averaging the signal present between 260 and 300 ms after an offer is presented (MFN). We identify reliable average MFNs to offers on three scalp locations (Fz, FC1, and Cz in the 10/20 EEG system, see Fig. 1A and Fig. S1). MFNs seem to be more pronounced, particularly in response to unfair offers in the UG compared to the IG (*p* = 0.007, Table S2.6, Fig. 1B). This suggests that in the IG responders decide to accept any offer already before it has been presented, rendering expectation processing of the offers obsolete. The response is stronger for prosocial responders (*p* < 0.001, Fig. 1C, Tables S2.6, S2.7), which is in line with that those with stronger social preferences are more concerned about unfair offers in the UG^19,20^.

We also evaluate the P2, an event-related positivity on medial-frontal electrode locations preceding the MFN averaging the signal between 180 and 220 ms following the offer. When assessing the baseline effects of the P2 we see higher amplitudes in response to fair compared to unfair offers only in the UG for prosocial responders (*p* < 0.001, see Fig. 1A–C and Tables S2.8, S2.9). This is in line with earlier research showing that the P2 is most pronounced in response to fair offers in prosocial responders^19^.

For the EMG measures, we focus on the corrugator supercilii, or ‘frown’ muscle, located medially above the eyes, and the zygomaticus major, or ‘smile’ muscle, located lateral to the nose. In a trial-by-trial regression model, we find that unfair offers only evoke a very slight increase in frown (*p* = 0.047) over all games, but also that this frowning contrast between fair and unfair offers is stronger in prosocial than in individualistic individuals (interaction effect *p* = 0.002), and is especially strong for prosocials in the IG (three-way interaction *p* = 0.020, see Fig. 1E and Table S2.10). This is not only in line with prior work showing that prosocial responders are more emotionally affected by unfairness^19^ but also suggests that this effect is most pronounced when equality cannot be restored. Effects on the zygomaticus are small and do not differ significantly between games and types of participants, although there is a slightly increased smile when responders receive an unfair offer (*p* = 0.041, see Fig. 1E and Table S2.11). These smile effects do not seem to permit unambiguous interpretation though may perhaps indicate mild disbelief.

### Explaining rejection behavior in UG

We now turn to our final question about what predicts rejection behavior. Because we have hardly any rejections in the IG, we do not have enough rejection data to perform this analysis in the IG. We therefore restrict our attention to rejection in the UG. In two-level logistic regression analysis of rejection behavior in the UG, we control for being prosocial and self-reported anger given that these are the relevant predictors of rejection behavior found across all responders. As biophysical predictors we use FAA, trial-by-trial EMG based responses (corrugator and zygomaticus), and the averaged MFN difference between fair and unfair UG-offers (unfair minus fair). Averaged MFNs were used to ensure that this predictor has a reliable signal-to-noise ratio. All biophysical measures are evaluated as main effect as well as in interaction with social value orientation.

First, we find that prosocial responders are more likely to reject unfair offers in the UG than individualistic responders (Table S1.5, 46% vs. 27%, *p* = 0.001; Table S2.13, *p* = 0.003). This result replicates earlier results^16,19,25^, but not from Yamagishi et al.^8^, who do not find a relation with social value orientation. We find a net increase of 35 percentage points in the probability of rejection as a result of being prosocial (Table S2.13). Second, we find that facial muscle reactions following offers, which in our earlier reporting we found to respond to fairness, seem not to predict rejection. Third, our evidence suggests that the fairness effect in the MFN predicts rejection on electrode-locations Fz (positively) and FC1 (negatively) for prosocial participants, while they do not for individualists (Table S2.13). The effects interact with being prosocial in the underlying regression analysis (respectively *p* = 0.004 and *p* = 0.003). The opposite direction of these predictors reflects a shift of the MFN to unfair offers to a more left-sided scalp-location when the subsequent response is to reject. Figure 1D visualizes this effect by suggesting that rejected compared to accepted unfair offers not only show an amplified MFN, but also that this MFN is shifted to the left-hemisphere. This MFN-shift to the left relatively decreases the MFN on the central location (Fz) while amplifying it on the lateral location (FC1), hence this can explain their opposite relation with rejection behavior. When controlling for P2 activity all effects remain of similar magnitude (see Table S2.14), which suggests that the MFN effects on predicting rejection are due to expectancy processing and not due to preceding attention processing. For individualists, we also observe that MFN amplitude, on location Cz, predicts rejection, but this effect disappears after controlling for P2 amplitudes suggesting this is not due to expectancy but attentional processing. Finally, we observe some associations with post hoc self-reported emotions (Table S.2.14), but they are based on uncontrolled between-subject comparisons in post-treatment self-report measures, which are the weakest form of evidence. Although the effect of self-reported anger on rejection within individualists could be a residual effect of emotions after preference differences and game differences are controlled, it might also be a reverse-causality effect that individualists post hoc report more anger to reflect their discontent with rejecting offers against their preferences.

Together these results extend the findings by^7,17,19,26^, to a trial-by-trial prediction of rejection based on averaged MFN reactions. Importantly, these results suggest that this is exclusively the case in prosocial responders, further supporting the social preferences-based account of rejection behavior. Lastly, we see that a more right-sided FAA predicts rejection behavior negatively for prosocial responders (*p* = 0.028, Table S2.13). In other words, prosocial responders who are more prone to approach motivation, aggression, and anger do not reject unfair offers more often. Thus, again emotions do not seem to be a major driver of rejection.

## Discussion

Altogether these results clearly come down on the side of social preferences as an explanation for why people take no deal over a bad deal. Although participants exhibited *stronger* feelings of anger after unfair offers when the proposer had impunity, they nonetheless rejected these offers much *less often*. Moreover, expectancy processing and not facially expressed anger predicted rejection of unfairness, particularly in individuals that experience a mismatch of unfairness with what they expect due to their prosocial preferences. This combination implies that participants controlled their emotions and took the cost of punishment and its effect on the proposer into account in their decision.

We draw this conclusion on the basis of a design that overcame persistent limitations of prior experimental work. Previous studies seeking to differentiate between the preference-based account and the emotion-based account either did not control proposer behavior or did so through automation, removing the interpersonal element from the interaction. We achieved a human-to-human setup in which proposers and responders were in the same room and directly interacting, while still ensuring that differences in proposer behavior could not be the cause of differences in responder behavior across the two games. We combined this setup with a combination of facial expression and brain activity measures. Although we do find that unfair offers trigger corrugator (‘frown’) activity, this expression of emotion does not trigger rejection behavior. This makes our interpretation plausible that social preferences trigger both behavior and emotions. Crucially, the elevated medial-frontal brain reactions to unfairness that we find suggest that it is expectancy processing rooted in the social preference of the responder that accounts for rejection behavior. Indeed, the MFN and associated ACC activity have been consistently linked to fairness concerns and encountering norm violations, not only in the EEG literature, but also in the body of fMRI studies as shown in a recent meta-analysis^27,28^.

A limitation of the study is that having participants play real games with present opponents came at the cost of the number of trials we could run. Because unfair proposals were made a little over 40%, we had only around 20 unfair offers per participant and these were not equally distributed among all the participants. This reduced the power of the EEG analysis and prohibited more detailed analyses and comparisons of objective emotion measurements over time for different events such as comparing emotions after rejecting or accepting unfair offers. Such analyses might have shed more light on how certain actions affected the emotional stress that participants incurred after unfair experiences.

Another limitation is that several of our results are merely correlational. The difference in rejection rates between IG and UG can be causally related to the within-subject experimentally varied games, and the differences in rejection behavior, MFN measures, and EMG measures between fair and unfair offers can be considered causal consequences of these offers. However, the relations between self-reports on emotions, MFN measures, EMG measures, and rejection behavior are all correlational. We indicated this issue already for the subjective measurements, but also the direct causal links between brain activity and facial reaction after unfair offers and the subsequent behavior need further investigation.

Our study was not designed to differentiate between types of social preferences as explanations for rejection behavior. Nonetheless, our findings provide some clues as to whether reciprocity or inequity aversion drove participants’ behavior. A relevant feature of the emotion measurements is that the subjective responses at the end as well as a continuous frown in the corrugator supercilii indicate that anger does not easily disappear after the rejection of the unfair offer. This suggests that the inequity aversion explanation is less plausible because arguably the rejection should imply that the proposer and responder are even again and the anger should become less. In addition, if inequity aversion would have been an important driver, envy should have been as strongly related to behavior as anger, which we did not find to be the case (Table S2.5). This is also consistent with findings by^29^ indicating that participants are not so much concerned with equality, but with the violation of expected behavior and with^6^ showing that if participants have other means to express their concerns, getting equal through rejection becomes less important. Taken together, this tentatively suggests that it is a preference for reciprocation rather than for equity that leads people to choose no deal over a bad deal.

A recent study^30^ shows that, although anger is mildly related to rejection behavior, low-arousal negative emotions such as disappointment are more strongly related to rejection behavior. It is difficult to reconcile this result with ours because the result is based on self-reported emotions before rejection behavior, while in our case participants report emotions much later. Also, the proposals in this study are not made by actual other participants and are always unfair. Most importantly, the study cannot distinguish between prosocial and individualistic participants while particularly low-arousal emotions such as disappointment due to an unfair offer could be due to expectations of fairness driven by social preferences shown in our study. Thus, these results signal that it is worthwhile to consider other emotions in relation to social preferences when trying to explain rejection behavior.

Finally, we tried to exclude the role of communication explicitly from our design by not providing any game-level information on responder behavior or payoffs to proposers. Of course, this does not imply that we do not see a role in communication and that possibilities for communication might also relate to emotional reactions as found in other studies^31^. One could argue that even in our design, rejection behavior in the UG can be seen as a signal to the proposer because the proposer will receive less payoff by the end of the experiment. The option to give this signal could contribute to the difference in rejection rates between the UG and IG, because this signal is not possible in the IG in our setup. Still, we consider this potential effect of such a signal also a preference-related explanation in line with Bolton and Katok^9^ who consider the ‘symbolic punishment’ in the IG if the decision is communicated to the proposer as an indication of someone’s dissatisfaction with the unfair outcome.

## Methods

156 students were recruited using ORSEE^32^ from the ELSE Laboratory participant pool at Utrecht University. Participants were asked to send an email to the experiment leader if they wanted to be included as a participant for whom we would also do the biophysical measurements. These participants were paid an additional 25 euro show-up fee for extra time and discomfort. They were called and explained what the measurements exactly implied and it was checked whether they had no history of psychological illness, because then they could not be included. If participants indeed wanted to undergo the biophysical measurements, they were invited one hour earlier to the laboratory for installing EEG, EMG and skin conductance electrodes. The whole procedure including information and consent forms was in line with all guidelines and procedures for human experiments and the experimental protocols were approved by the Ethics Committee of the Faculty of Social and Behavioural Sciences, Utrecht University under case number FETC18-104.

The biophysical measures were obtained at 2048 Hz sampling rate using four Biosemi ActiveTwo (https://www.biosemi.com/products.htm) amplifiers synchronized in a daisy-chain which streamed the data to a single data-acquisition PC. EEG was recorded from 32 Ag/AgCl pin electrodes placed over the scalp according to the International 10/20 EEG system. EMG was recorded using 4 Ag/AgCl flat-type electrodes placed on the corrugator supercilii^2^ and zygomaticus major^2^ muscles. Electro-oculogram (EOG), to be sued for correcting the EEG data for eye movements^33^, was recorded using Ag/AgCl flat-type electrodes placed on the suborbital and supraorbital of the right eye and on the external canthi of both eyes. The ground consisted of the active common mode sense and passive driven right leg electrode. Skin conductance was measured using two passive Nihon Kohden electrodes attached to the hand, but due to the multiplicity of measurements and the fact that we did not want to impose a delay on the responses to the offers, we could not stick to the waiting times necessary to obtain reliable skin conductance responses^18^ and indeed these measured turned out to be too noisy to be used in the study. When participants arrived at the lab, they read a short information letter and signed the informed consent form. Two researchers installed all the measurement instruments and tested the signal quality. Before other participants were allowed into the laboratory, a resting state EEG was made for the four participants present, which consisted of 4 min sitting in a chair divided in four alternating 1-min intervals with eyes opened/closed.

In an adjacent room, the other participants also received the EEG information letter although they did not have any measurements themselves and signed the informed consent form. After the other participants were let into the laboratory, the general instructions on the UG and IG were handed out (see SI) and 48 rounds were played. Note that after all proposers had made their offers in a given round, there first appeared a cross on the screen of the responders and they were instructed to make sure to focus on the screen, because the actual offer would appear within a couple of seconds. This time was jittered between one and five seconds to prevent exact anticipation of when the offer would appear. The exact screens and sequences of these core trials are shown in section 4 of the SI. After all rounds were played, the participants obtained some further questions including subjective emotion indicators for responders in the different games and for the different offers as well as an incentivized social value orientation test using the six-item version of the slider measure^34^ using the z-Tree implementation of^35^. The implementation includes a calculation of division of participants that depended on the extent to which they value their own versus the other participant’s payoff. Exactly as described by Murphy et al.^34^, participants are classified in potentially four categories: altruistic, prosocial, individualistic, or competitive participants. In our experiment, there were only prosocial and individualistic responders and, therefore, we construct a dummy variable ‘prosocial’ to contrast these two groups in the analyses. We also included behavioral inhibition and behavioral activation scale^36^, moods^37^, and the Buss-Perry aggression scale^38^. These personal characteristics turn out to have no effect on behavior in our games and controlling for them did not change our results (analyses not shown). The experiment was implemented using z-Tree^39^. Task events (obtaining offers and expressing responses) were synchronized with the biophysical data by sending trigger signals over a parallel cable from each individual z-Tree PC to an in-house built trigger box, which in turn transferred the signals instantaneously to the Biosemi trigger box linked to the biophysical data-acquisition PC.

EEG data were processed using BrainVision Analyzer 2.1. Data were down-sampled to 256 Hz, re-referenced offline to the average activity of all 32 EEG locations, and 1–30 Hz band-pass filtered with a 48 dB roll-off per octave. Voltage steps > 50 µV/ms were marked as artifacts and the 400 ms interval surrounding the artifact was excluded from further analyses.

Resting-state EEG data were divided in four 1-min segments of which the two segments containing eyes-closed data were further segmented in sixty 2-s intervals. A fast Fourier transform (Hamming window: length 10%) was used to estimate spectral power (μV^2^) in the alpha frequency band (8–12 Hz) and averaged across all sixty segments. Frontal alpha-asymmetry was computed by subtracting the natural logarithm of average left-sided alpha-power (locations: F3, FC1, FC5, and C3) from the natural logarithm of average right-sided alpha-power (locations: F4, FC2, FC6, and C4)^22^.

Event-related EEG data were corrected for eye movements based on the EOG data using the Gratton and Coles algorithm^32^ as implemented in BrainVision Analyzer 2.1, segmented based on the trigger-signals synchronized to the on-screen appearance of the offers (− 100 to 1000 ms relative to the trigger-signal) and baseline-corrected based on the 100 ms preceding the trigger-signal. Waveforms were first averaged across all trials to identify the location of the MFN (FC1, Fz, and Cz, see Fig. S1). Next, averages were computed for the separate trial conditions: game type (IG and UG) by offer (fair and unfair). For further analyses, MFN amplitudes for these conditions were scored by computing the average signal in the separate three target electrodes in the interval 260–300 ms after the offer. For visualization purposes we also computed average waveforms for fair and unfair trials across the two games (see Fig. 1A) and for accepted and rejected offers in the UG (see Fig. 1D). P2 amplitudes were also scored for the same electrode-locations and task-conditions by computing average signal in the interval 180–220 ms after the offer.

EMG data were also processed in BrainVision Analyzer 2.1. For both muscles data from the two attached electrodes were subtracted from each other, 30–500 Hz band-pass and 50 Hz notch filtered with a 48 dB roll-off per octave, rectified, segmented based on the trigger-signals synchronized to the on-screen appearance of the offers (− 1000 to 4000 ms relative to the trigger-signal), and baseline-corrected based on the 1000 ms preceding the trigger-signal. We also segmented the data based on the trigger-signals synchronized with the response (0 to 2000 ms relative to the trigger-signal) and these segments were baseline-corrected using the same baseline as the offer-segments. For further analyses of EMG reactions to offers and responses we computed the averages across the 2000 ms following the trigger-signal for offer and response as well as 2000–4000 ms following the offer.

All statistical analyses are based on logistic (for decisions) or linear (for biophysical measures) two-level regression analyses with the interactions as units of analyses and random effects at the level of participants. Tables with average marginal effects based on these regressions can be found in the SI. EMG measures are constructed by calculating mean values over two seconds; for emotion measures of respondents measured after offers, we consider the two seconds after the offer appeared and we have done robustness checks with a four-second period. No results depend on whether we use the two- or four-second intervals (results not reported). Because of the strong difference between rejection rates in the UG and the IG, we examine emotional explanations for UG and IG for the two types of games separately. We do not predict rejection in the IG for participants with biophysical measures only, because in that case we have only 24 rejections spread over 8 participants left. Reliable estimations are impossible given that this event is so rare.

## Supplementary Information

Supplementary Information.

## Data Availability

The datasets used and/or analyzed during the current study are available from the corresponding author on reasonable request.
