# Supplementary material for: Social preferences trump emotions in human responses to unfair offers
Source: Sci Rep. 2023 Jun 13;13:9602. doi: 10.1038/s41598-023-36715-y (PMC10264406; doi:10.1038/s41598-023-36715-y)
Supplement: Supplementary file 1 — Supplementary Information. [file 41598_2023_36715_MOESM1_ESM.docx]

ug_ig_sr_si_2023_04_25

**Social Preferences Trump Emotions in Human Responses to Unfair Offers**

**Supporting Information**

**Vincent Buskens, Ingrid Kovacic, Elwin Rutterkamp,**

**Arnout van de Rijt, David Terburg**

**Utrecht University**

**Table of Contents**

[1. Summary statistics 2](#_Toc133309599)

[2. Analyses of responder behavior 4](#_Toc133309600)

[3. Experiment instructions 13](#_Toc133309601)

[4. Description of a typical trial 16](#_Toc133309602)

# Summary statistics

**Participant statistics**

156 participants enrolled in the experiment; 78 as proposers and 78 as responders. The last group consisted of 38 participants without biophysical measurements and 40 with biophysical measurements. Each session had exactly four participants with biophysical measurements. The first session consisted of 18 participants, the subsequent six of 16 participants, and the last three sessions had 14 participants. 52 participants identified as male (33.3%) and 104 as female; the average age of the participants was about 24.6 years (st. dev. 6.86). Note that one page was accidentally omitted in the final questionnaire given to participants in the first two sessions leading to missing values on the variable for age for 34 participants. Based on the social value orientations slider measure (Murphy et al. 2011), we classify participants are either prosocial, individualistic, or competitive (Murphy et al (2011: 773). Of the 78 proposers, 40 are classified as prosocial based on their answers on the social value orientation slider measure, 37 as individualistic, and 1 as competitive. Of the 78 responders, 27 are classified as prosocial and 51 as individualistic.

**Proposer behavior**

The deliberate design decision to not inform proposers whether they play a UG or an IG achieved comparable levels of fair offers in UG and IG. As reported in Table S1.1, the proportion of unfair offers is around 0.42. The difference between UG and IG is not significant (*p* = 0.085 in a two-level logistic regression model with random effects at the participant level and the game being the only predictor). The percentage of unfair offers made by proposers was stable throughout the experiment and did not differ between contiguous blocks of 12 IG / UG games. As Table S1.2 shows, prosocial participants proposed unfair offers in 34% of the games, while individualistic proposers did this in 51% of the games, which is a significant difference (*p* = 0.025) in a two-level logistic regression model.

**Table S1.1.** Proposer behavior in offers

| Game | Unfair | Fair | Total |
| --- | --- | --- | --- |
| IG | 802  42.84 | 1070  57.16 | 1872 |
| UG | 768  41.03 | 1104  58.97 | 1872 |
| Total | 1570  41.93 | 2174  58.07 | 3744 |

**Table S1.2.** Proposer behavior by type of proposer

| Game | Unfair | Fair | Total |
| --- | --- | --- | --- |
| Individualists | 903  50.84 | 873  49.16 | 1776 |
| Prosocials | 647  33.70 | 1273  66.30 | 1920 |
| Competitors | 20  41.67 | 28  58.33 | 48 |
| Total | 1570  41.93 | 2174  58.07 | 3744 |

**Responder behavior**

Responders rejected 0% of fair offers, while they rejected 33% of unfair offers in the UG and 6% in the IG (see Tables S1.3 and S1.4). Responders received offers in four blocks of 12 games. Each block consisted of either 12 UG games or 12 IG games. Over the course of the 48 games the responders played, they alternated between UG and IG blocks. Half of the responders started with 12 UG games and the other half with 12 IG games. Rejection rates of unfair offers for the UG do not differ significantly between UG games played in blocks 1, 2, 3, or 4. Although there are very few rejections in the IG, they tend to become even smaller after an experience with the UG. In the first block, when participants start with 12 IGs, the rejection rate is 14.5%; for participants who experienced one series of UGs (playing IGs in blocks 2 or 3), the rejection rate is about 5% and for participants who experienced two series of UGs (playing IGs in block 4), the rejection rate is only 1.5%. As Table S1.5 shows, prosocials reject unfair offers more often in the UG than individualistic participants (*p* = 0.002) in a two-level logistic regression.

**Table S1.3.** Rejection of fair offers

| Game | Accept | Reject | Total |
| --- | --- | --- | --- |
| IG | 1070  100.00 | 0  0.00 | 1072 |
| UG | 1102  99.82 | 2  0.18 | 1104 |
| Total | 2172  99.91 | 2  0.09 | 2174 |

**Table S1.4.** Rejection of unfair offers

| Game | Accept | Reject | Total |
| --- | --- | --- | --- |
| IG | 750  93.52 | 52  6.48 | 802 |
| UG | 512  66.67 | 256  33.33 | 768 |
| Total | 1262  80.38 | 308  19.62 | 1570 |

**Table S1.5.** Rejection of unfair offers in UG by prosocial and individualistic responders

| Game | Accept | Reject | Total |
| --- | --- | --- | --- |
| Individualists | 378  73.11 | 139  26.89 | 517 |
| Prosocials | 134  53.39 | 117  46.61 | 251 |
| Total | 512  66.67 | 256  33.33 | 768 |

**Participant earnings for the UGs and IGs**

For each game, 100 points were divided, together worth 0.40 euros. Proposers earned on average 2740 points over the course of the 48 games they played. Always offering the fair offer, and always having this accepted, would have earned them 2400 points. The overall acceptance rate of 80% also shows that the expected value of an unfair offer was about 0.8 times 80 is 64 points, which is larger than the 50 points that can be expected from a fair offer. Responders without EEG earned on average 1695 points, while responders with EEG earned 1736 points. This difference between responders with and without EEG is not significant (*p* = 0.417).

# Analyses of responder behavior

Unfair offers in the UG are more often rejected than unfair offers in the IG. Rejection rates do not differ between responders with or without connection to biophysical measurement instruments, indicated by the dummy variable EEG (see Table S2.1).

**Table S2.1.** Average marginal effects in two-level logistic regression models predicting rejection of unfair offers with random effects for responders (*N* = 1570 in 78 respondents)

|  | Model 1 | | | Model 2 | | |
| --- | --- | --- | --- | --- | --- | --- |
|  | **Coef.** | **St. err.** | ***p*-value** | **Coef.** | **St. err** | ***p*-value** |
| UG | 0.257 | 0.028 | 0.000 | 0.280 | 0.036 | 0.000 |
| EEG |  |  |  | 0.011 | 0.064 | 0.868 |
| UG × EEG |  |  |  | -0.043 | 0.036 | 0.227 |
|  |  |  |  |  | | |
| Loglikelihood | -455.846 | | | -455.086 | | |

**Subjective emotional ratings and difference tests**

Responders score generally low on anger and envy with the highest average being 1.56 on a 0 to 4 scale (not at all, a little, moderately, quite a lot, extremely) for unfair offers in the IG. In terms of happiness, responders display somewhat starker differences, again with the lowest happiness for unfair offers in the IG. Fair offers imply less anger, less envy, and more happiness than unfair offers, both in the IG as well as in the UG, all differences being strongly significant, *p* < 0.001. Unfair offers in the IG lead to more anger, more envy, and less happiness than unfair offers in the UG, again for all differences *p* < 0.001 (see Table S2.2). The reason does not seem to be that participants dislike the IG less in general, because anger, envy, and happiness ratings for fair offers are comparable between the IG and the UG (see Table S2.2). Prosocials differ more in dissatisfaction with unfair offers between UG and IG than individualists. Based on two-sample t-tests, this difference is significantly larger for anger (*p* = 0.016) implying that prosocials are relatively angrier receiving unfair offers in the IG than in the UG compared to individualists. These differences are not significantly larger for envy (*p* = 0.581) or happiness (*p* = 0.526).

**Table S2.2.** Summary statistics for subjective emotion ratings and paired *t*-test results for differences between ratings for UG and IG (78 participants)

| Emotion | Mean | Standard  deviation | Paired *t*-test, *p*-value |
| --- | --- | --- | --- |
| Anger (UG, unfair) | 0.99 | 0.99 | 0.000 |
| Anger (IG, unfair) | 1.56 | 1.32 |  |
| Envy (UG, unfair) | 0.90 | 0.92 | 0.000 |
| Envy (IG, unfair) | 1.76 | 1.22 |  |
| Happy (UG, unfair) | 0.91 | 1.06 | 0.000 |
| Happy (IG, unfair) | 0.32 | 0.67 |  |
| Anger (UG, fair) | 0.15 | 0.51 | 0.096 |
| Anger (IG, fair) | 0.31 | 0.71 |  |
| Envy (UG, fair) | 0.33 | 0.75 | 0.213 |
| Envy (IG, fair) | 0.46 | 0.83 |  |
| Happy (UG, fair) | 2.31 | 1.26 | 0.056 |
| Happy (IG, fair) | 2.01 | 1.30 |  |

**Table S2.3.** Summary statistics for subjective emotion ratings and paired *t*-test results for differences in ratings between UG and IG among prosocials (27 participants)

| Emotion | Mean | Standard  deviation | Paired *t*-test, *p*-value |
| --- | --- | --- | --- |
| Anger (UG, unfair) | 0.89 | 0.97 | 0.000 |
| Anger (IG, unfair) | 1.85 | 1.29 |  |
| Envy (UG, unfair) | 0.78 | 0.80 | 0.000 |
| Envy (IG, unfair) | 1.74 | 1.16 |  |
| Happy (UG, unfair) | 1.04 | 0.90 | 0.000 |
| Happy (IG, unfair) | 0.33 | 0.68 |  |
| Anger (UG, fair) | 0.04 | 0.19 | 0.025 |
| Anger (IG, fair) | 0.44 | 0.85 |  |
| Envy (UG, fair) | 0.26 | 0.71 | 0.010 |
| Envy (IG, fair) | 0.74 | 1.16 |  |
| Happy (UG, fair) | 2.67 | 1.18 | 0.029 |
| Happy (IG, fair) | 2.04 | 1.32 |  |

**Table S2.4.** Summary statistics for subjective emotion ratings and paired *t*-test results for differences in ratings between UG and IG among individualists (51 participants)

| Emotion | Mean | Standard  deviation | Paired *t*-test, *p*-value |
| --- | --- | --- | --- |
| Anger (UG, unfair) | 1.04 | 1.00 | 0.007 |
| Anger (IG, unfair) | 1.41 | 1.31 |  |
| Envy (UG, unfair) | 0.96 | 0.98 | 0.000 |
| Envy (IG, unfair) | 1.76 | 1.26 |  |
| Happy (UG, unfair) | 0.84 | 1.14 | 0.005 |
| Happy (IG, unfair) | 0.31 | 0.68 |  |
| Anger (UG, fair) | 0.22 | 0.61 | 0.851 |
| Anger (IG, fair) | 0.24 | 0.62 |  |
| Envy (UG, fair) | 0.37 | 0.77 | 0.627 |
| Envy (IG, fair) | 0.31 | 0.55 |  |
| Happy (UG, fair) | 2.12 | 1.28 | 0.666 |
| Happy (IG, fair) | 2.06 | 1.30 |  |

**Predicting rejecting of unfair offers using self-reported emotions and social value orientations**

Rejecting unfair offers turns out to be related to self-reported anger and social value orientations (Note: gender and self-reported behavioral inhibition, behavioral activation, and aggression do not predict rejecting unfair offers in the UG or the IG. Decision time does not predict rejection in the UG, but has a positive effect on rejection in the IG.) As expected and reported in Table S2.5, prosocial participants reject unfair offers more in the UG also when controlling for subjective emotions. In addition, only individualistic participants who reported being angrier rejected more (the anger evaluation for the unfair outcome in the analyzed game is used in the analysis), but angrier prosocial participants do not reject more than less angry prosocial participants (the interaction effect between being prosocial and anger is also significant in the analysis (*p* = 0.006)). A 1-point increase on the anger scale increases the rejection rate by about 15% in the UG for individualists. Although there is a very weak effect of happiness for individualists in this analysis suggesting that individualists who feel happier with an unfair offer reject this offer more often, we put less emphasis on this finding given that the effect is only marginally significant and not robust over different model specifications. There is also a weak negative effect of envy for prosocials on rejection in the IG, but also this effect is not robust and in the logistic regression no effects are significant. That we do not find strong effects for the IG is not surprising given the low number of rejections. Generally, we interpret the effects reported in Table S2.5 with caution, as they rely exclusively on uncontrolled between-subject comparisons.

**Table S2.5.** Average marginal effects based on two-level logistic regression models predicting rejection of unfair offers in the UG and IG separately using social value orientations and self-reported emotion measures (random effects at the level of 78 participants). Average marginal effects for self-reported emotion measures are reported for prosocial and individualistic participants separately.

|  | UG | | | IG | | |
| --- | --- | --- | --- | --- | --- | --- |
|  | **Coef.** | **St. err.** | ***p*-value** | **Coef.** | **St. err** | ***p*-value** |
| Prosocial (vs. individualist) | 0.246 | 0.082 | 0.003 | 0.045 | 0.040 | 0.259 |
| Effects for prosocials |  |  |  |  |  |  |
| Anger | -0.049 | 0.077 | 0.526 | 0.052 | 0.037 | 0.161 |
| Envy | -0.051 | 0.092 | 0.577 | -0.074 | 0.035 | 0.036 |
| Happiness | 0.004 | 0.082 | 0.966 | -0.021 | 0.064 | 0.739 |
|  |  |  |  |  |  |  |
| Effects for individualists |  |  |  |  |  |  |
| Anger | 0.154 | 0.032 | 0.000 | 0.013 | 0.015 | 0.420 |
| Envy | -0.013 | 0.040 | 0.734 | 0.011 | 0.017 | 0.557 |
| Happiness | 0.066 | 0.032 | 0.039 | -0.055 | 0.067 | 0.410 |
|  |  | | |  | | |
| Loglikelihood | -294.110 | | | -115.74 | | |
| Number of decisions | 768 | | | 802 | | |

**Baseline EEG and EMG analyses**

**Identification of relevant MFN locations**

Relevant MFN electrodes were identified from a CSD map in the interval 260-300 ms after the offer across all responders and trials (see Fig. S1) and used for subsequent analyses that are all orthogonal to the contrast used for electrode identification.


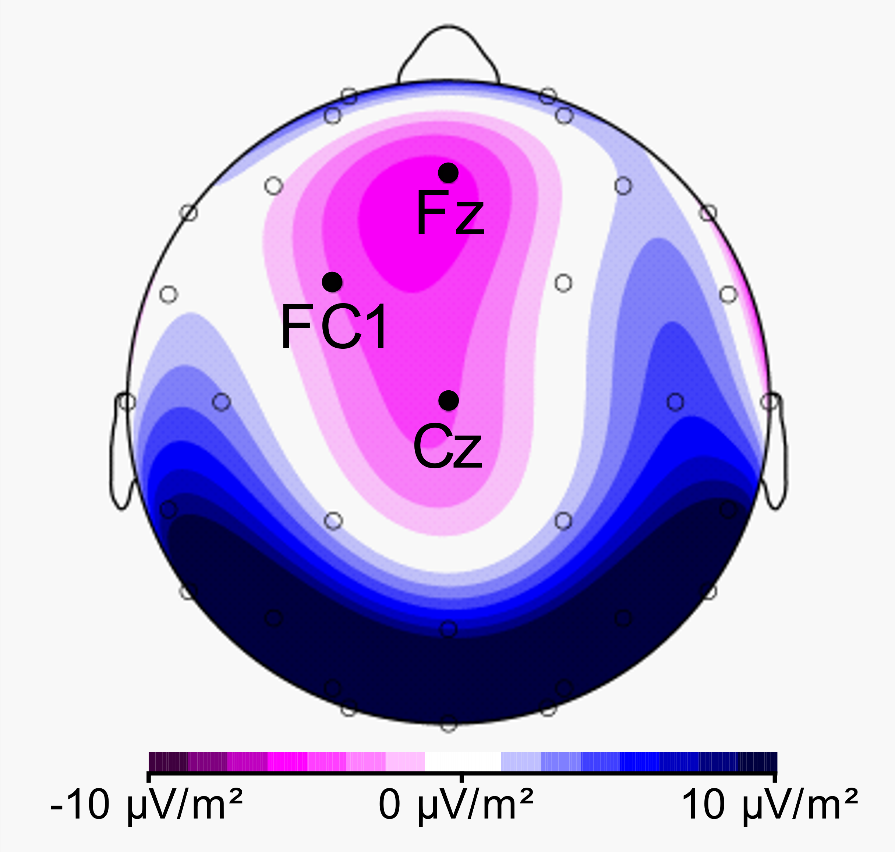


Figure S1: CSD map in the interval 260-300 ms after the offer across all responders and trials

**Relating EEG amplitudes after offers to game, offer and social value orientations**

After determining the average MFN per participant, offer, and game as explained in the methods part of the paper, we run two-level logistic regression models to determine the relationships between being prosocial, offer, and game and the amplitudes of the three relevant electrodes: FC1, Fz, and Cz. What exactly is included in the fixed parts of the various models is explicated in the table captions. We add random intercepts for participants and random slopes at the participant level for game and offer and their interaction as well if both variables are included (Tables S2.6 and S2.8). We assume random slopes to be independent, because adding correlations does not improve the models. We include only a random slope for offer in analyses that focus on the UG (Tables S2.7 and S2.9).

We report relevant average marginal effects of receiving an unfair compared to a fair offer corresponding to results reported in Figure 1 and the accompanying text in the paper. The effects are averaged over all the observations that are implied by what is indicated in brackets in each row of the table. In each table, we start with the overall comparison and then zoom in on smaller subsets of observations.

**Table S2.6.** Average marginal effects of receiving an unfair offer from a two-level logistic regression model predicting MFN of FC1, Fz, Cz combined using the electrodes, game, and offers as predictors as well as all two- and three-way interactions. Random intercepts for participants are included as well as independent random slopes for game, offer and the interaction (453 observations nested in 38 participants)

| Comparison | Contrast | Std. error | *p*-value | Figure |
| --- | --- | --- | --- | --- |
| Unfair vs Fair (overall) | -0.460 | 0.279 | 0.099 |  |
| Unfair vs Fair (IG) | 0.119 | 0.301 | 0.693 |  |
| Unfair vs Fair (UG) | -1.032 | 0.381 | 0.007 |  |
| Unfair vs Fair (FC1) | -0.379 | 0.360 | 0.293 | Figure 1, panel A, FC1 |
| Unfair vs Fair (Fz) | -0.290 | 0.360 | 0.421 | Figure 1, panel A, Fz |
| Unfair vs Fair (Cz) | -0.711 | 0.360 | 0.048 | Figure 1, panel A, Cz |
| Unfair vs Fair (FC1 in IG) | 0.277 | 0.442 | 0.531 | Figure 1, panel B, FC1, IG |
| Unfair vs Fair (Fz in IG) | -0.128 | 0.442 | 0.771 | Figure 1, panel B, Fz, IG |
| Unfair vs Fair (Cz in IG) | 0.209 | 0.442 | 0.636 | Figure 1, panel B, Cz, IG |
| Unfair vs Fair (FC1 in UG) | -1.027 | 0.497 | 0.039 | Figure 1, panel B, FC1, UG |
| Unfair vs Fair (Fz in UG) | -0.449 | 0.497 | 0.367 | Figure 1, panel B, Fz, UG |
| Unfair vs Fair (Cz in UG) | -1.620 | 0.497 | 0.001 | Figure 1, panel B, Cz, UG |

Table S2.6 shows that the MFN is more negative if participants receive unfair offers in the UG, but not in the IG. In the model the interaction between game and offer is significant (*p* = 0.003) showing that indeed the MFN is stronger (more negative) for unfair offers than for fair offers, both in the UG and in the IG. Table S2.6 as well as Figure 1 in the paper show that the effect is especially seen for electrodes FC1 and Cz.

**Table S2.7.** Average marginal effects of receiving an unfair offer from a two-level logistic regression model predicting MFN of FC1, Fz, Cz combined in the UG using the electrodes, game, and social value orientations as predictors as well as all two- and three-way interactions. Random intercepts for participants are included as well as a random slope for offer (228 observations nested in 38 participants)

| Comparison | Contrast | Std. error | *p*-value | Figure |
| --- | --- | --- | --- | --- |
| Unfair vs Fair (individualists) | -0.228 | 0.344 | 0.507 |  |
| Unfair vs Fair (prosocials) | -2.410 | 0.450 | 0.000 |  |
| Unfair vs Fair (FC1 for individualists) | -0.117 | 0.552 | 0.832 | Figure 1, panel C, FC1, ind. |
| Unfair vs Fair (Fz for individualists) | 0.591 | 0.552 | 0.284 | Figure 1, panel C, Fz, ind. |
| Unfair vs Fair (Cz for individualists) | -1.158 | 0.552 | 0.036 | Figure 1, panel C, Cz, ind. |
| Unfair vs Fair (FC1 for prosocials) | -2.586 | 0.723 | 0.000 | Figure 1, panel C, FC1, pro. |
| Unfair vs Fair (Fz for prosocials) | -2.232 | 0.723 | 0.002 | Figure 1, panel C, Fz, pro. |
| Unfair vs Fair (Cz for prosocials) | -2.411 | 0.723 | 0.001 | Figure 1, panel C, Cz, pro. |

Table S2.7 shows that the MFN in the UG is more negative after unfair offers than after fair offers for prosocial participants but not for individualistic participants. Table S2.7 as well as Figure 1 in the paper show that prosocial participants have stronger MFN on all electrodes after unfair offers than after fair offers, while individualists only have a significant effect for electrode Cz.

**Table S2.8.** Average marginal effects of receiving an unfair offer from a two-level logistic regression model predicting P2 of FC1, Fz, Cz combined using the electrodes, game, and offers as predictors as well as all two- and three-way interactions. Random intercepts for participants are included as well as independent random slopes for game, offer and the interaction (453 observations nested in 38 participants)

| Comparison | Contrast | Std. error | *p*-value | Figure |
| --- | --- | --- | --- | --- |
| Unfair vs Fair (overall) | -0.244 | 0.293 | 0.404 |  |
| Unfair vs Fair (IG) | 0.210 | 0.302 | 0.485 |  |
| Unfair vs Fair (UG) | -0.693 | 0.415 | 0.094 |  |
| Unfair vs Fair (FC1) | -0.262 | 0.368 | 0.477 | Figure 1, panel A, FC1 |
| Unfair vs Fair (Fz) | -0.130 | 0.368 | 0.725 | Figure 1, panel A, Fz |
| Unfair vs Fair (Cz) | -0.342 | 0.368 | 0.354 | Figure 1, panel A, Cz |
| Unfair vs Fair (FC1 in IG) | 0.279 | 0.438 | 0.524 | Figure 1, panel B, FC1, IG |
| Unfair vs Fair (Fz in IG) | 0.216 | 0.438 | 0.662 | Figure 1, panel B, Fz, IG |
| Unfair vs Fair (Cz in IG) | 0.136 | 0.438 | 0.756 | Figure 1, panel B, Cz, IG |
| Unfair vs Fair (FC1 in UG) | -0.796 | 0.521 | 0.126 | Figure 1, panel B, FC1, UG |
| Unfair vs Fair (Fz in UG) | -0.471 | 0.521 | 0.366 | Figure 1, panel B, Fz, UG |
| Unfair vs Fair (Cz in UG) | -0.813 | 0.521 | 0.118 | Figure 1, panel B, Cz, UG |

Table S2.8 shows that there are no significant differences for P2 between fair and unfair offers in either UG or IG if we do not distinguish between individualistic and prosocial participants.

**Table S2.9.** Average marginal effects of receiving an unfair offer from a two-level logistic regression model predicting P2 of FC1, Fz, Cz combined in the UG using the electrodes, game, and social value orientations as predictors as well as all two- and three-way interactions. Random intercepts for participants are included as well as a random slope for offer (228 observations nested in 38 participants)

| Comparison | Contrast | Std. error | *p*-value | Figure |
| --- | --- | --- | --- | --- |
| Unfair vs Fair (individualists) | -0.011 | 0.334 | 0.975 |  |
| Unfair vs Fair (prosocials) | -1.864 | 0.438 | 0.000 |  |
| Unfair vs Fair (FC1 for individualists) | -0.323 | 0.575 | 0.574 | Figure 1, panel C, FC1, ind. |
| Unfair vs Fair (Fz for individualists) | 0.550 | 0.575 | 0.339 | Figure 1, panel C, Fz, ind. |
| Unfair vs Fair (Cz for individualists) | -0.259 | 0.575 | 0.652 | Figure 1, panel C, Cz, ind. |
| Unfair vs Fair (FC1 for prosocials) | -1.607 | 0.753 | 0.033 | Figure 1, panel C, FC1, pro. |
| Unfair vs Fair (Fz for prosocials) | -2.222 | 0.753 | 0.003 | Figure 1, panel C, Fz, pro. |
| Unfair vs Fair (Cz for prosocials) | -1.763 | 0.753 | 0.019 | Figure 1, panel C, Cz, pro. |

Table S2.9 shows that in the UG the P2 is stronger after fair than after unfair offers, but only for prosocial individuals, indicating that fair offers draw more attention, particularly in prosocial responders, consistent with prior research (19). This pattern is, because of their opposite polarity, analogous to what we observed for prosocials on the MFN, which makes it important that we check whether effects of MFN persist after controlling for P2 as we do in Table S2.13.

**Table S2.10.** Average marginal effects of receiving an unfair offer from a two-level logistic regression model predicting EMG corrugator activity (frown) using the game, offer, and social value orientations as well as all two- and three-way interactions. Random intercepts for participants are included as well as independent random slopes for game, offer and the interaction (1920 observations nested in 40 participants)

| Comparison | Contrast | Std. error | *p*-value | Figure |
| --- | --- | --- | --- | --- |
| Unfair vs Fair (overall) | 0.091 | 0.046 | 0.047 |  |
| Unfair vs Fair (individualists) | 0.019 | 0.058 | 0.750 |  |
| Unfair vs Fair (prosocials) | 0.214 | 0.076 | 0.005 |  |
| Unfair vs Fair (IG) | 0.044 | 0.065 | 0.494 |  |
| Unfair vs Fair (UG) | 0.140 | 0.065 | 0.032 |  |
| Unfair vs Fair (individualists. in IG) | -0.111 | 0.082 | 0.175 | Figure 1, panel E, corru. |
| Unfair vs Fair (prosocials in IG) | 0.302 | 0.105 | 0.004 | Figure 1, panel E, corru. |
| Unfair vs Fair (individualists in UG) | 0.148 | 0.082 | 0.069 | Figure 1, panel E, corru. |
| Unfair vs Fair (prosocials in UG) | 0.125 | 0.108 | 0.244 | Figure 1, panel E, corru. |

Table S2.10 shows that participants frown more, especially prosocial participants, if they receive unfair offers rather than fair offers. The largest effect occurs for prosocial participants in the IG, suggesting that they are especially angry with an unfair offer if they cannot punish the proposer. Note that in the original model the main effect of UG (*p* = 0.038), the interaction effect offer times prosocial (*p* = 0.002), and the three-way interaction offer times UG times prosocial (*p* = 0.020) are significant.

**Table S2.11.** Average marginal effects of receiving an unfair offer from a two-level logistic regression model predicting EMG zygomaticus activity (smile) using the game, offer, and social value orientations as well as all two- and three-way interactions. Random intercepts for participants are included as well as independent random slopes for game, offer and the interaction (1920 observations nested in 40 participants)

| Comparison | Contrast | Std. error | *p*-value | Figure |
| --- | --- | --- | --- | --- |
| Unfair vs Fair (overall) | 0.065 | 0.032 | 0.041 |  |
| Unfair vs Fair (individualists) | 0.079 | 0.040 | 0.048 |  |
| Unfair vs Fair (prosocials) | 0.041 | 0.052 | 0.432 |  |
| Unfair vs Fair (IG) | 0.026 | 0.045 | 0.557 |  |
| Unfair vs Fair (UG) | 0.103 | 0.045 | 0.021 |  |
| Unfair vs Fair (individualists in IG) | 0.067 | 0.057 | 0.238 | Figure 1, panel E, zygo. |
| Unfair vs Fair (prosocials in IG) | -0.042 | 0.072 | 0.565 | Figure 1, panel E, zygo. |
| Unfair vs Fair (individualists in UG) | 0.092 | 0.056 | 0.103 | Figure 1, panel E, zygo. |
| Unfair vs Fair (prosocials in UG) | 0.123 | 0.074 | 0.095 | Figure 1, panel E, zygo. |

Table S2.11 shows that participants seem to smile more after unfair offers than after fair offers in some of the conditions, even if effects are overall smaller than for the corrugator (S2.10). We speculate that some minimal smiling occurs out of disbelief because of receiving unfair offers.

**Predicting rejection of unfair offers using biophysical and self-report measures**

We now predict rejecting unfair offers in the UG with the biophysical measures. We have measures in the reaction in the corrugator supercilii (frowning) and zygomaticus major (smiling) from the EMG. We use the average reaction during 2 seconds after the offer (robustness checks have been done for only 4 seconds as well as with the reaction around the own response; all lead to the same substantive results). We use the ERP for the Fz, Cz, and FC1 electrodes (average valence from 260-300ms after the offer; for robustness, we also detected the peak value in the interval 260-340ms after the offer and the same result is found). We also control for the relevant self-reported measures: the social value orientation and the anger related to unfair offers in the UG as well as for frontal-alpha asymmetry (FAA) as a measure for trait anger (Table S2.13). We provide additional analyses to show that our results are also robust if we control for attention measured through P2 (Table S2.14).

Before we run the multivariate analysis we explore the correlations between the relevant measures for unfair offers in the UG (Table S2.12). Note that the EEG measures and the self-reports are measured at the individual level. So correlations are based on the 37 participants for which we have reliable EEG measurements. It turns out that self-reported anger and FAA are not significantly positively correlated. The high correlations between some of the electrodes are significant. This is expected given the relation between these signals described in the main text. Furthermore, only the correlation between FAA and Fz is weakly significant (*p* = 0.040), which is likely to be coincidental among so many correlations with only a limited number of cases. For the other measures, we also did not expect high correlations: FAA is trait anger measured independently of any offer, EMG measures are direct facial reactions on the unfair offers, while the self-reports are based on participants’ recollection of their anger over all unfair offers in the UG measured after all the trials have been finished.

**Table S2.12.** Correlations between self-reported anger, corrugator supercilii, zygomaticus major,

FAA (trait anger), FC1, Fz, Cz

|  | Self-reported anger | Corrugator supercilii | Zygomaticus major | FAA (trait anger) | FC1 | Fz |
| --- | --- | --- | --- | --- | --- | --- |
| Corrugator supercilii | -0.035 |  |  |  |  |  |
| Zygomaticus major | -0.051 | -0.075 |  |  |  |  |
| FAA (trait anger) | 0.231 | -0.077 | 0.013 |  |  |  |
| FC1 | 0.128 | -0.068 | -0.035 | 0.167 |  |  |
| Fz | 0.107 | -0.022 | -0.012 | 0.340 | 0.723 |  |
| Cz | 0.239 | -0.132 | -0.060 | 0.073 | 0.515 | 0.261 |

Results of the multivariate analyses (Table S2.13) show that prosocials are more likely to reject unfair offers. Among prosocials MSN strongly predicts rejection while only weakly among individualists. Corrugator and zygomaticus do not predict rejection. This suggests that rejection is based on expectation processing and not on emotional reactions. There are also unanticipated effects of self-reported anger, but we are reluctant to interpret these effects because they are based on uncontrolled between-subject comparisons and only in either prosocial or individualistic participants. For example, the effect of self-reported anger on individualists’ rejection could be a residual effect of emotions after preference differences and game differences are controlled, but it might also be a reverse-causality effect that individualist post-hoc report more anger to reflect their discontent with them acting against their preferences. The MFN effects continue to be strong once P2 measures are controlled (Table S2.14), suggesting that they are due to expectancy rather than attention processing.

**Table S2.13.** Average marginal effects based on two-level logistic regression models predicting rejection of unfair offers in the UG using self-report anger, biophysical measures, being prosocial and interactions of all other measures with being prosocial (random intercepts are included at the participant level, random slopes are excluded, because random slopes for corrugator and zygomaticus were estimated 0 in a model with random slopes; N = 366 within 37 participants). Average marginal effects are reported for prosocials and individualists separately.

|  | Coef. | St. err. | *p*-value |
| --- | --- | --- | --- |
| Prosocial | 0.369 | 0.060 | 0.000 |
|  |  |  |  |
| Effects prosocials |  |  |  |
| Self-reported anger | -0.005 | 0.079 | 0.951 |
| Corrugator supercilii | -0.047 | 0.048 | 0.324 |
| Zygomaticus major | 0.030 | 0.029 | 0.302 |
| FAA (trait anger) | -2.017 | 0.917 | 0.028 |
| FC1 | -0.290 | 0.111 | 0.009 |
| Fz | 0.306 | 0.095 | 0.001 |
| Cz | 0.049 | 0.049 | 0.320 |
|  |  |  |  |
| Effects individualists | | | |
| Self-reported anger | 0.121 | 0.054 | 0.026 |
| Corrugator supercilii | 0.016 | 0.022 | 0.447 |
| Zygomaticus major | -0.039 | 0.032 | 0.232 |
| FAA (trait anger) | -0.006 | 0.248 | 0.980 |
| FC1 | 0.049 | 0.032 | 0.123 |
| Fz | 0.020 | 0.023 | 0.374 |
| Cz | -0.068 | 0.022 | 0.002 |
|  |  |  |  |
| Loglikelihood | -137.070 | | |

**Table S2.14.** Average marginal effects based on two-level logistic regression models predicting rejection of unfair offers in the UG using self-reported anger, MFN, trait anger and P2, being prosocial and interactions of all other variables with being prosocial (random intercepts are included at the participant level; N = 366 within 37 participants; EMG measures are removed from the model to prevent an overload of variables, but including them leads to exactly the same results)

|  | Coef. | St. err. | *p*-value |
| --- | --- | --- | --- |
| Prosocial | 0.350 | 0.055 | 0.000 |
|  |  |  |  |
| Effects prosocials |  |  |  |
| Self-reported anger | -0.050 | 0.102 | 0.623 |
| FAA (trait anger) | -2.665 | 1.269 | 0.036 |
| FC1 (MFN) | -0.359 | 0.149 | 0.016 |
| Fz (MFN) | 0.398 | 0.140 | 0.004 |
| Cz (MFN) | 0.068 | 0.104 | 0.513 |
| FC1 (P2) | 0.043 | 0.090 | 0.632 |
| Fz (P2) | -0.073 | 0.055 | 0.180 |
| Cz (P2) | 0.035 | 0.061 | 0.564 |
|  |  |  |  |
| Effects individualists | | | |
| Self-reported anger | 0.191 | 0.059 | 0.001 |
| FAA (trait anger) | -0.302 | 0.235 | 0.119 |
| FC1 (MFN) | -0.023 | 0.040 | 0.570 |
| Fz (MFN) | 0.024 | 0.030 | 0.430 |
| Cz (MFN) | -0.012 | 0.034 | 0.727 |
| FC1 (P2) | 0.013 | 0.049 | 0.783 |
| Fz (P2) | 0.035 | 0.029 | 0.223 |
| Cz (P2) | -0.065 | 0.034 | 0.055 |
|  |  |  |  |
| Loglikelihood | -132.455 | | |

# Experiment instructions

**- Instructions -**

**The experiment**

Welcome to this experiment. Please read the following instructions carefully. These instructions are the same for all participants. The instructions state everything you need to know in order to participate in the experiment. If you have any questions, please raise your hand. One of the experimenters will approach you and answer your question.

Please turn off your phone and put it in your bag. During the experiment you are not allowed to communicate with other participants. You may also not do any other tasks, because they will distract you from the experiment. Thank you very much.

Some participants are connected to EEG measurement instruments. These participants are involved in exactly the same tasks as everyone else and interact with the other participants in the lab. They have been invited to come earlier to the experiment to prepare the measurements. In addition, it is important for the measurements that the experiment runs in a rather regular speed. Therefore, please stay concentrated, answer questions consciously, but do not take more time than necessary.

You can earn money by means of earning points during the experiment. The number of points that you earn depends on your own choices and the choices of other participants. At the end of the experiment, the total number of points that you earned will be exchanged at an exchange rate of:

**100 points = 0.4 Euro**

The money you earn will be paid out in cash at the end of the experiment. Other participants will not see how much you have earned. Participants without EEG receive an additional 5 Euro show-up fee. Participants with EEG will receive a 30 Euro show-up fee because of the extra time and burden they undergo during this experiment.

**The decision situation**

First, we introduce the decision situation. All participants will be assigned to a role A or B. Half of the participants will be a so-called person A and the other half will be a person B.

The decision situation is as follows. First, person **A** receives 100 points and proposes how to divide this amount between A and B. Person A can choose between two offers:

1. persons A and B both receive 50 points;
2. person A keeps 80 points and offers 20 points to person B.

See the computer screen below.


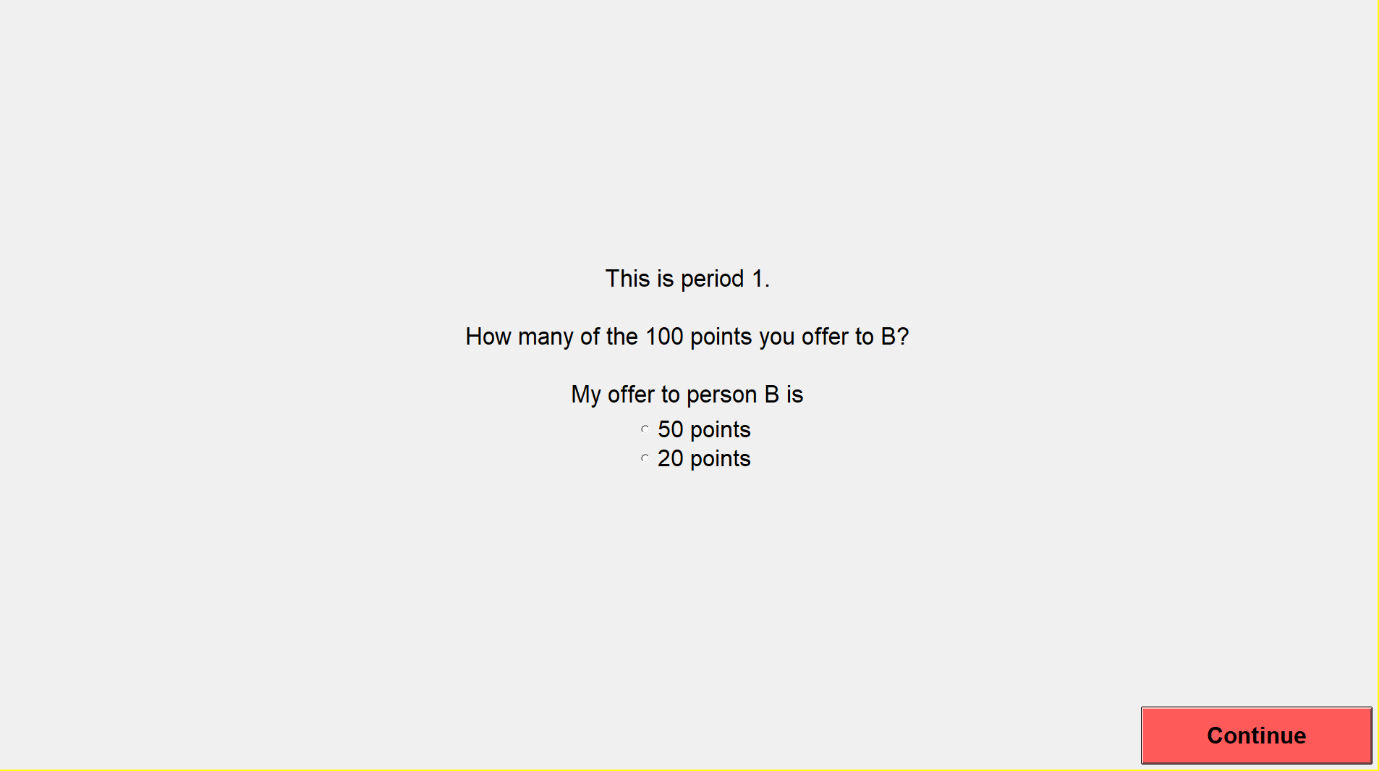


Second, person **B** chooses to **accept** or **reject** the offer of **A**. See the screen below.


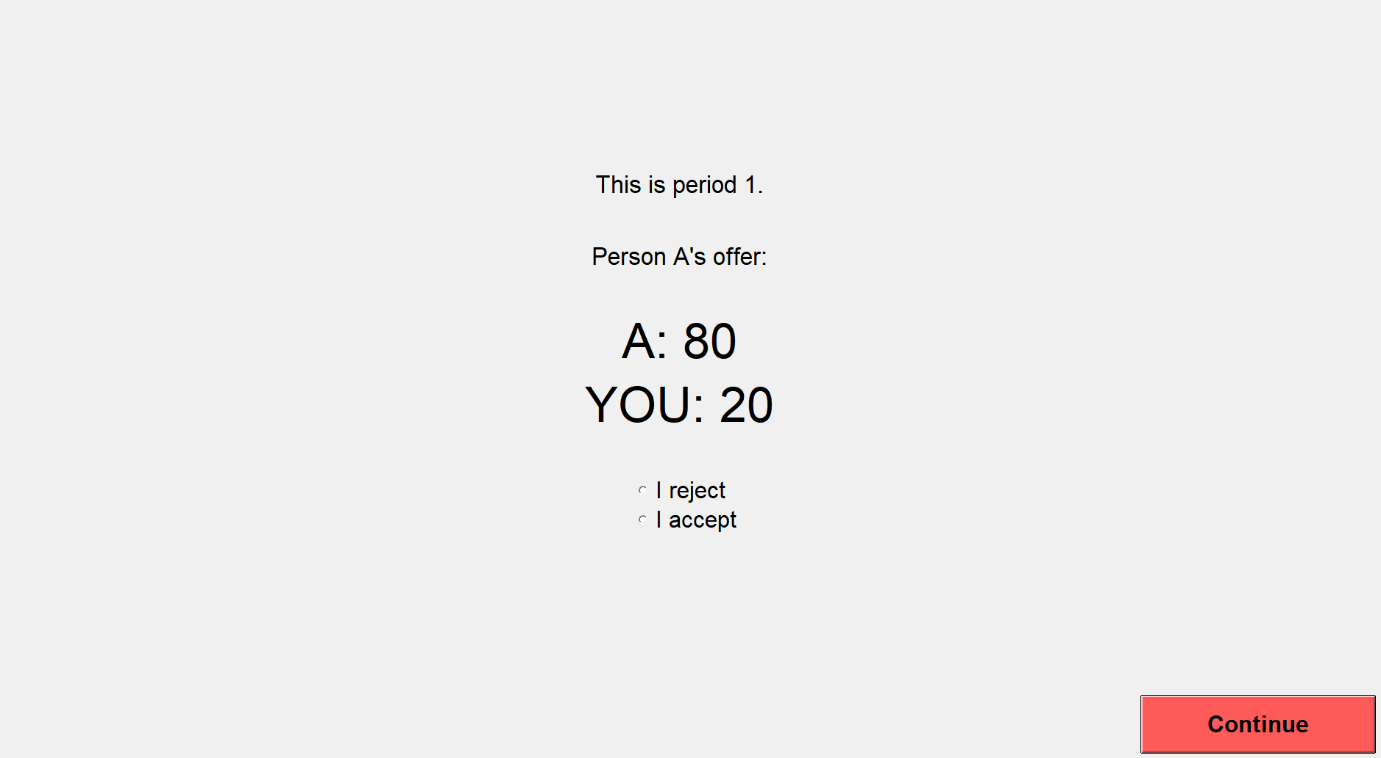


If **B** **accepts** the offer, the money will be allocated as it was proposed by person **A**.

If **B rejects** the offer, there are two possibilities:

1. A keeps the amount he/she proposed to keep, while B receives nothing;
2. A and B both receive nothing.

**IMPORTANT: person A does not know whether he or she does or does not receive the share if B rejects the offer, but person B does know whether person A keeps his or her share.**

The decision situation will now be repeated for 48 periods. In each period again, each person **A** is anonymously and randomly paired with a person **B**.

- You as well as all other participants are in the same role throughout these 48 periods.
- From period to period, your partner changes.
- You will never be informed with which other participant you were matched in which period.

In every period, Person **A** has a 50/50 chance whether he or she keeps the share if B rejects the offer.

Half of the persons B will play first 12 periods in which **A** does not keep his/her share if B rejects. The other half of persons B start with 12 periods in which A does keep his/her share. Persons B switch conditions every 12 periods. They are informed about this via explicit messages on their computer screens.

After every period, the points you earned are added to your account.

Note that persons A are not informed about the decisions of persons B during the 48 periods.

All participants are informed about how many points they earned in total over the 48 periods at the end of the 48 periods.

It is important for the EEG measurement that person **B** concentrates on the screen when the offer appears. The offers of persons **A** will only appear after all persons **A** have chosen. Therefore, we will show a big “+” on the screen a couple of seconds before the actual offer appears. The “+” thus signals that you need to concentrate on the offer to appear.

After the 48 periods, there will be a few additional choices in which you can earn some more points and which will be explained on the screen. The experiment will end with a questionnaire. Please also fill in the questionnaire in a concentrated manner.

After you have finished reading these instructions, please press the continue button on the screen and the experiment begins. If you have any questions please raise your hand and one of the experimenters will approach you.

# Description of a typical trial

A typical try starts with all proposers seeing the following screen, while the proposers see a screen asking them to wait.


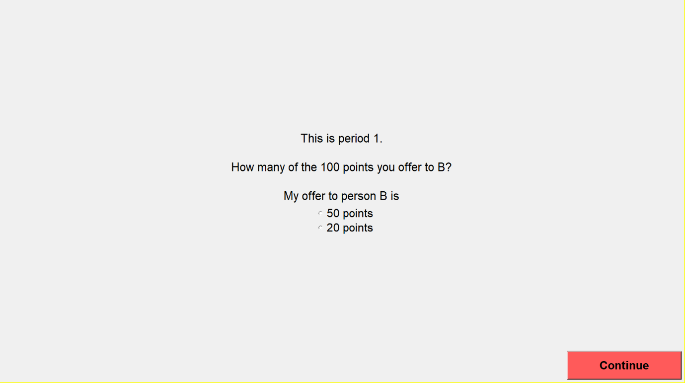


All proposers have 30 seconds to respond and if they do not, they are nudged to make a decision. In earlier periods, proposers take somewhat more time than after they get some experience. After all proposers have made their choices the responders are shown the below attention cross.


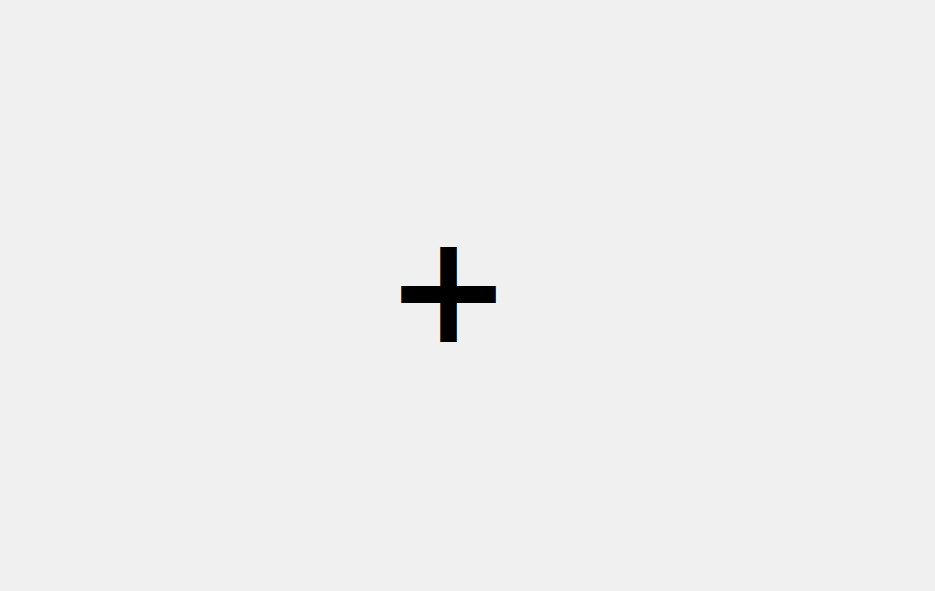


The responders are informed that after some seconds after the cross appears they will see the offer of the proposer and so they should pay attention. The actual time until the appearance of the offer is randomized and can be any time between one and five seconds. At this time, the proposers see a screen asking them to wait. After the attention period has finished, the responders are shown the following screen.


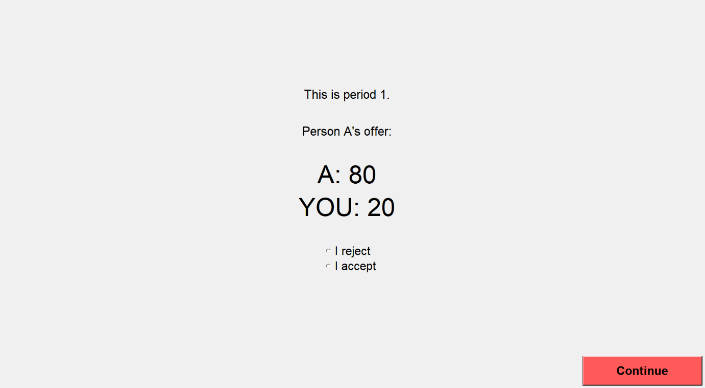


All responders also have 30 seconds to respond. After they all responded, proposers are shown the first screen again. The total trial takes between one minute at the start of the experiment to about 20 seconds when participants have more experience.
